# Navigating the fungal battlefield: cysteine-rich antifungal proteins and peptides from Eurotiales

**DOI:** 10.3389/ffunb.2024.1451455

**Published:** 2024-09-03

**Authors:** Jeanett Holzknecht, Florentine Marx

**Affiliations:** Biocenter, Institute of Molecular Biology, Innsbruck Medical University, Innsbruck, Austria

**Keywords:** antifungal proteins and peptides, fungal pathogens, fungal infection, antifungal resistance, structure-function relation, antifungal mode of action

## Abstract

Fungi are ubiquitous in the environment and play a key role in the decomposition and recycling of nutrients. On the one hand, their special properties are a great asset for the agricultural and industrial sector, as they are used as source of nutrients, producers of enzymes, pigments, flavorings, and biocontrol agents, and in food processing, bio-remediation and plant growth promotion. On the other hand, they pose a serious challenge to our lives and the environment, as they are responsible for fungal infections in plants, animals and humans. Although host immunity opposes invading pathogens, certain factors favor the manifestation of fungal diseases. The prevalence of fungal infections is on the rise, and there is an alarming increase in the resistance of fungal pathogens to approved drugs. The limited number of antimycotics, the obstacles encountered in the development of new drugs due to the poor tolerability of antifungal agents in patients, the limited number of unique antifungal targets, and the low species specificity contribute to the gradual depletion of the antifungal pipeline and newly discovered antifungal drugs are rare. Promising candidates as next-generation therapeutics are antimicrobial proteins and peptides (AMPs) produced by numerous prokaryotic and eukaryotic organisms belonging to all kingdom classes. Importantly, filamentous fungi from the order Eurotiales have been shown to be a rich source of AMPs with specific antifungal activity. A growing number of published studies reflects the efforts made in the search for new antifungal proteins and peptides (AFPs), their efficacy, species specificity and applicability. In this review, we discuss important aspects related to fungi, their impact on our life and issues involved in treating fungal infections in plants, animals and humans. We specifically highlight the potential of AFPs from Eurotiales as promising alternative antifungal therapeutics. This article provides insight into the structural features, mode of action, and progress made toward their potential application in a clinical and agricultural setting. It also identifies the challenges that must be overcome in order to develop AFPs into therapeutics.

## 1 Introduction

### 1.1 The importance of fungi - insights into their benefits

**Figure 1:**
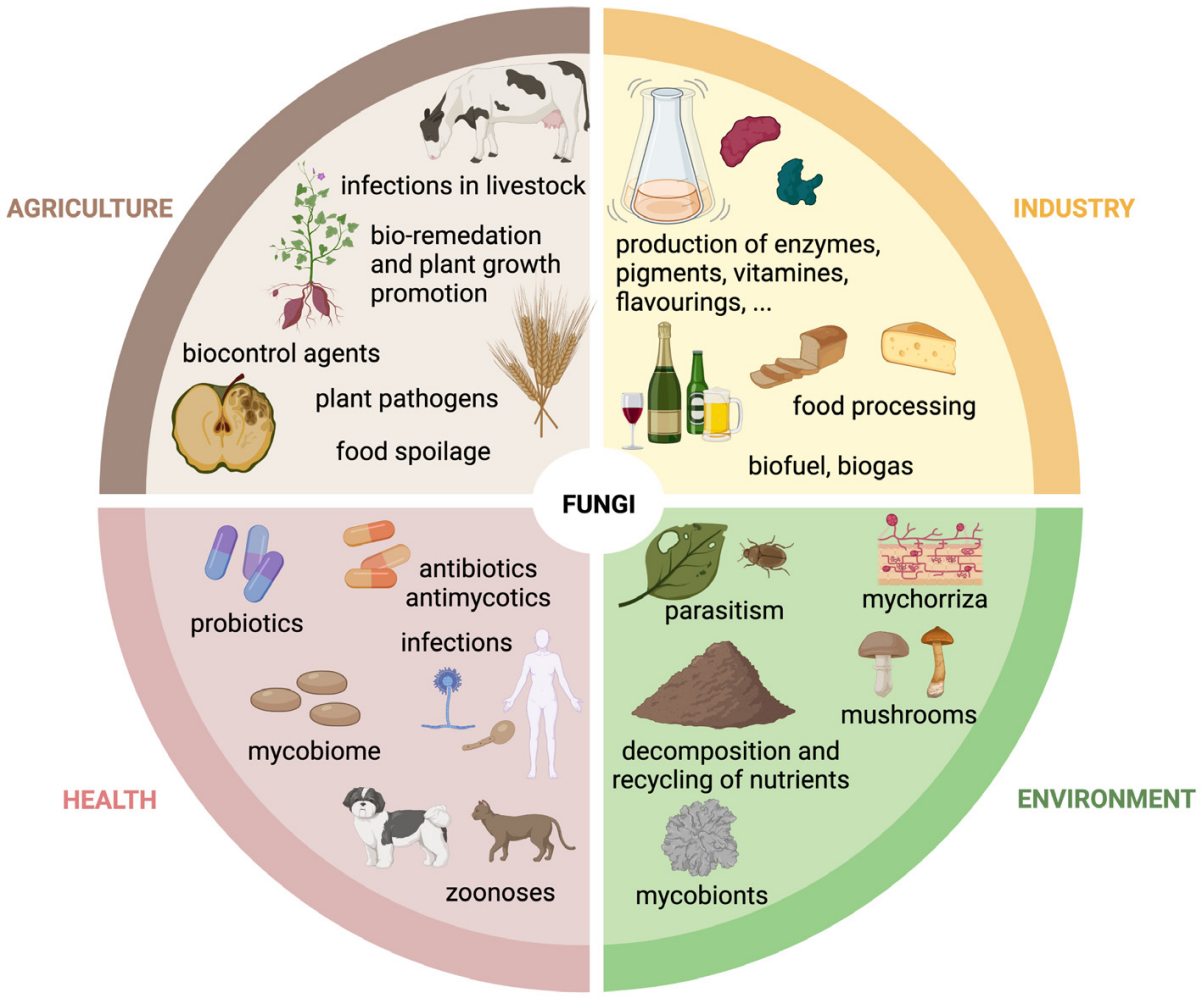
The role of fungi in agriculture, industry, health and environment. Schematic overview of the multifunctional roles of fungi in the agricultural, industrial, environmental and health sector. Created with BioRender.com.

The fungal kingdom comprises ∼6 million species, which are ubiquitous in the biosphere. Fungi exist in a wide variety of life forms, from microscopic single cells to the largest, documented organism on Earth (Sipos et al., 2018). Fungi have a substantial impact on our daily lives due to their integral role in the environment, industry, agriculture, and health. Accordingly, we benefit from positive properties or encounter negative aspects associated with them (**Figure 1**).

Fungal organisms are of fundamental importance in the environment and are classified into three groups based on their ecological functions: saprobic, symbiotic and parasitic fungi. Saprobic fungi contribute greatly to the well-being of the biosphere by fulfilling an important role as decomposers and recyclers of organic material. They secrete extracellular enzymes and enable maintenance and formation of soil structures and the recycling of nutrients into food webs for other organisms, which would otherwise be not available (O'Hanlon, 2017). Symbiotic fungi are characterized by their mutual relationships in which both partners gain advantages through their cooperation. A well-known example is mycorrhizal symbiosis, where a fungal partner interacts with the root system of a plant, providing nutrients, especially nitrates and phosphates, and receiving photosynthesis products, e.g. carbohydrates, in return (van der Heijden et al., 2015). Parasitic fungi are often perceived negatively, but they also fulfill important functions in their ecological niche, promoting biodiversity in the forest or landscape through natural selection (O'Hanlon, 2017).

Humans use fungi as a food source, e.g. in the form of mushrooms, as an alternative protein source for vegan diets, or as dietary supplements that have probiotic effects when consumed and contribute to the human well-being (Szajewska et al., 2016; Sen and Mansell, 2020; Grossmann and Weiss, 2021). The ability of fungi to excrete enzymes and metabolites is used in industry. Fungi, which belong to the division Ascomycota are widely used in this regard. Their key role is to support fermentation processes, enabling the production of a variety of fermented goods, such as alcoholic beverages, soy sauce, cheese, cured meat and bread (Dupont et al., 2017). Furthermore, they are exploited for their ability to secrete organic acids, lipids, enzymes, polysaccharides, amino acids, vitamins, polyols and pigments (Berovic and Legisa, 2007; Archer et al., 2008; Dufossé et al., 2014; Park et al., 2017; Raveendran et al., 2018). Fungi are also employed in the production of biogas and biofuel through the decomposition of biomass, and are utilized in waste treatment (Li et al., 2015; Jain et al., 2017; Passoth and Sandgren, 2019). Finally, the fungal production of biopharmaceuticals for healthcare applications represents a very important sector in the so-called “mycoindustry”. A prominent example is the β-lactam antibiotic penicillin, which is produced in large-scale by cultivating *Penicillium chrysogenum* under stress conditions in fermenters (Brakhage et al., 2004).

The ability of fungi to infect other organisms is used in agriculture for biological control, either against arthropods and nematodes, or even fungi (Pell et al., 2010; Thambugala et al., 2020). Due to rising problems with soil pollution, the remediation of environmental pollutants or chemical toxins through fungi, known as “mycoremediation”, is an increasingly utilized approach (Czaplicki et al., 2016; Akhtar and Mannan, 2020).

Although fungi are an integral part of the ecosystem, and their use is of great benefit to humankind, they have also taken on the role of major pathogens, promoted by significant ecological adaptations and evolutionary transitions.

### 1.2 The impact of fungi on health

Fungi can have negative effects on animal and human health in various ways. Passive health impairments can occur through the consumption of poisonous cap fungi, known as mycetism, which can lead to liver failure and death if the symptoms are not treated (Smith and Davis, 2016). Additionally, health can be impaired through the ingestion of contaminated food and feed. This can lead to the accumulation of mycotoxins in humans and animals. Mycotoxins are secondary metabolites produced by fungi, and are considered to be mutagenic, carcinogenic, and genotoxic. They enter the food chain and ultimately reach the end consumer via contaminated plant or animal products (Alshannaq and Yu, 2017; Khan, 2024; Zhang et al., 2024). Fungal spores are ubiquitous as aerosols and are constantly inhaled, which may cause allergic reactions and asthma (Hughes et al., 2022).

However, fungi can also directly harm the health by colonizing, spreading, and secreting metabolites that destroy the host's tissues. Fungi can act as pathogens if they meet four requirements: (i) adaptation to the host environment, ii) ability to penetrate host barriers, (iii) digestion and absorption of host tissue components, and (iv) evasion of the immune system. These criteria are met by either primary fungal pathogens, which rely solely on infecting the host for survival and may infect also immunocompetent individuals, or by opportunistic pathogens, which coexist with the host as harmless commensals and only cause infection under specific circumstances and predisposing conditions (Köhler et al., 2017).

Fungal infections are typically classified as either superficial or invasive. Superficial infections in humans for example can target the skin, hair, and nails, affecting ∼25% of the global population (Bongomin et al., 2017; Urban et al., 2020). In most cases these infections do not pose a significant threat to life, but frequently recur and are challenging to treat, which can have a detrimental impact on the psychosocial well-being of the patient.

**Figure 2:**
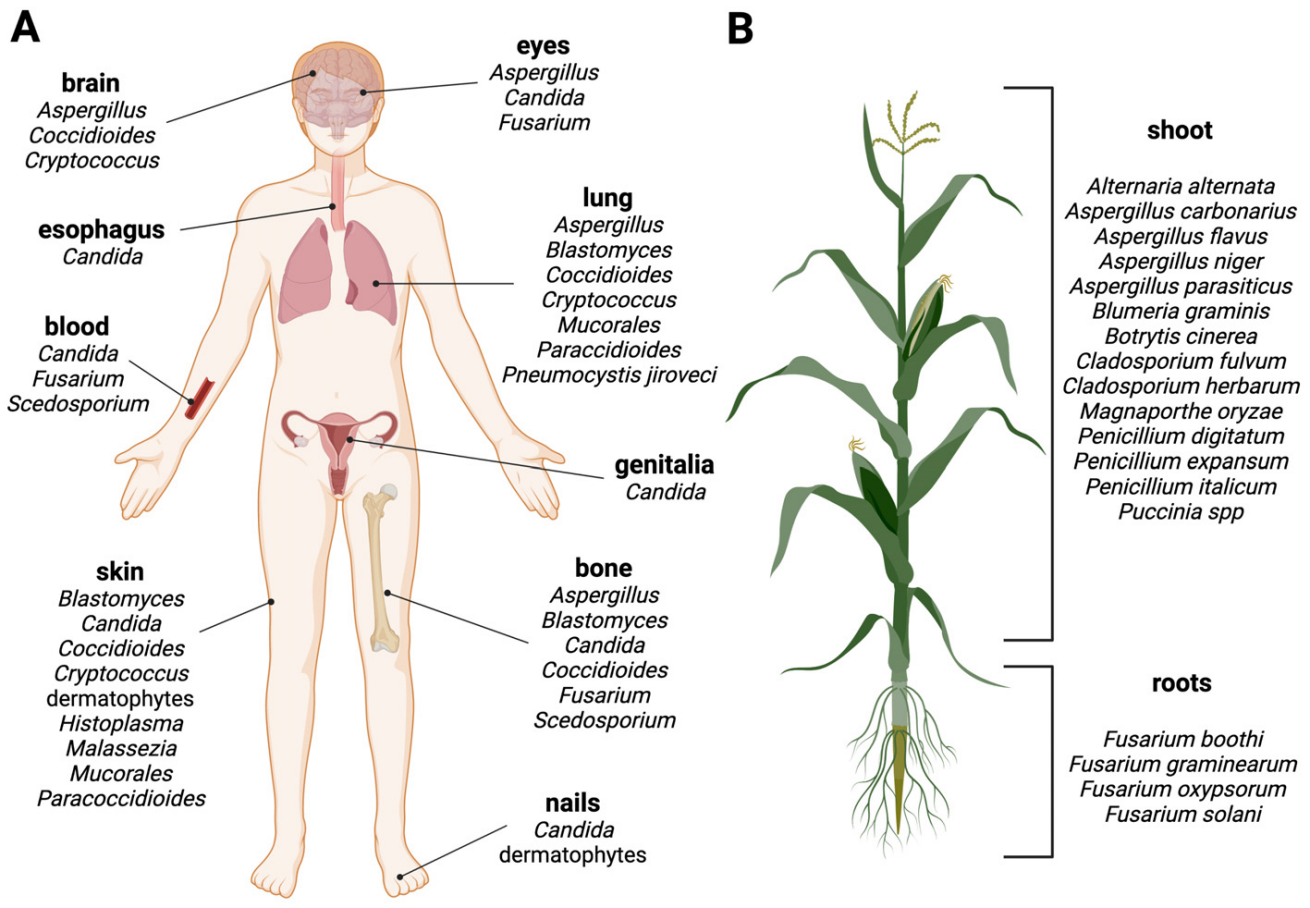
Fungal diseases in humans and plants. Schematic overview of fungal infection sites in **(A)** humans and **(B)** plants. Examples of human or plant pathogenic fungi are grouped by their predominant manifestation site. Created with BioRender.com.

Advanced medical interventions, including chemotherapy, organ transplants, invasive surgery, and immunosuppressive therapies, as well as underlying diseases affecting the host's immunity (e.g., AIDS, diabetes) has resulted in the emergence of a susceptible patient population with a compromised health status that is particularly vulnerable to fungal colonization of deeper tissues and organs, such as the lungs, bones, and the brain. Invasive fungal infections have the potential to be life-threatening (**Figure 2A**). Fungi are sometimes called "hidden killers" due to their often-neglected impact (Brown et al., 2012). A recent study estimated that over 6.5 million individuals are affected annually by life-threatening fungal diseases. Of these, more than 50% (3.7 million) succumb to the infection, with ∼68% of these deaths directly attributable to a fungal disease (Denning, 2024).

Fungal pathogens are typically not transmitted through personal contact in humans, with the exception of *Candida auris*, a multi-resistant yeast, that can cause healthcare associated outbreaks through nosocomial transmission (Tharp et al., 2023). Infections can be caused by the contact with fungi that are present in our environment, either through direct contact with contaminated objects (e.g., *Candida* spp. *via* medical devices, implants etc.), animals, soil, or plants (e.g., dermatophytes), or inhalation of fungal spores (e.g., *Aspergillus* spp.) to give some examples. However, in most cases infections are caused by a dysbiosis of the microbial species belonging to the body flora, also known as the microbiota. Many different microorganisms colonize the bodies of animals and humans as commensals without causing disease. For example, humans are the habitat for ∼100 fungal species, referred as mycobiota, that regulate each other's growth. However, when the balance in the body is disturbed, certain species from the host mycobiota cross the fine line between commensal and pathogenic lifestyles, overgrow others and lead to infections, *Candida albicans* being one of the most prominent examples of an opportunistic human pathogenic yeast (d'Enfert et al., 2021; Santus et al., 2021; Belvoncikova et al., 2022).

Phytopathogenic fungi pose a threat to plant health. They can have a broad or highly specialized host range, infecting specific plant tissues for reproductive life cycles and dispersal (**Figure 2B**) (Fisher et al., 2012). They can cause significant economic losses, destroying up to one-third of all food crops annually and thus posing a risk to the global food supply. Plant pathogenic fungi pose a threat in two ways: they can infect and destroy plants and crops, or they can produce toxic metabolites, known as mycotoxins, which contaminate plant products and processed foods of infected plant origin, leading to food and feed spoilage (Dean et al., 2012; Philippe, 2016; Savary et al., 2019).

## 2 The antifungal era on the brink of the abyss?

Fungal infections are prevented and treated with antifungal drugs. However, developing antifungal agents to combat fungal infections in the medical and agricultural fields has proven to be challenging due to the high similarity between fungal and host cells. It is difficult to determine a drug that is exclusively effective against fungi but harmless to plants, animals or humans (Perfect, 2017; Wall and Lopez-Ribot, 2020).

**Figure 3:**
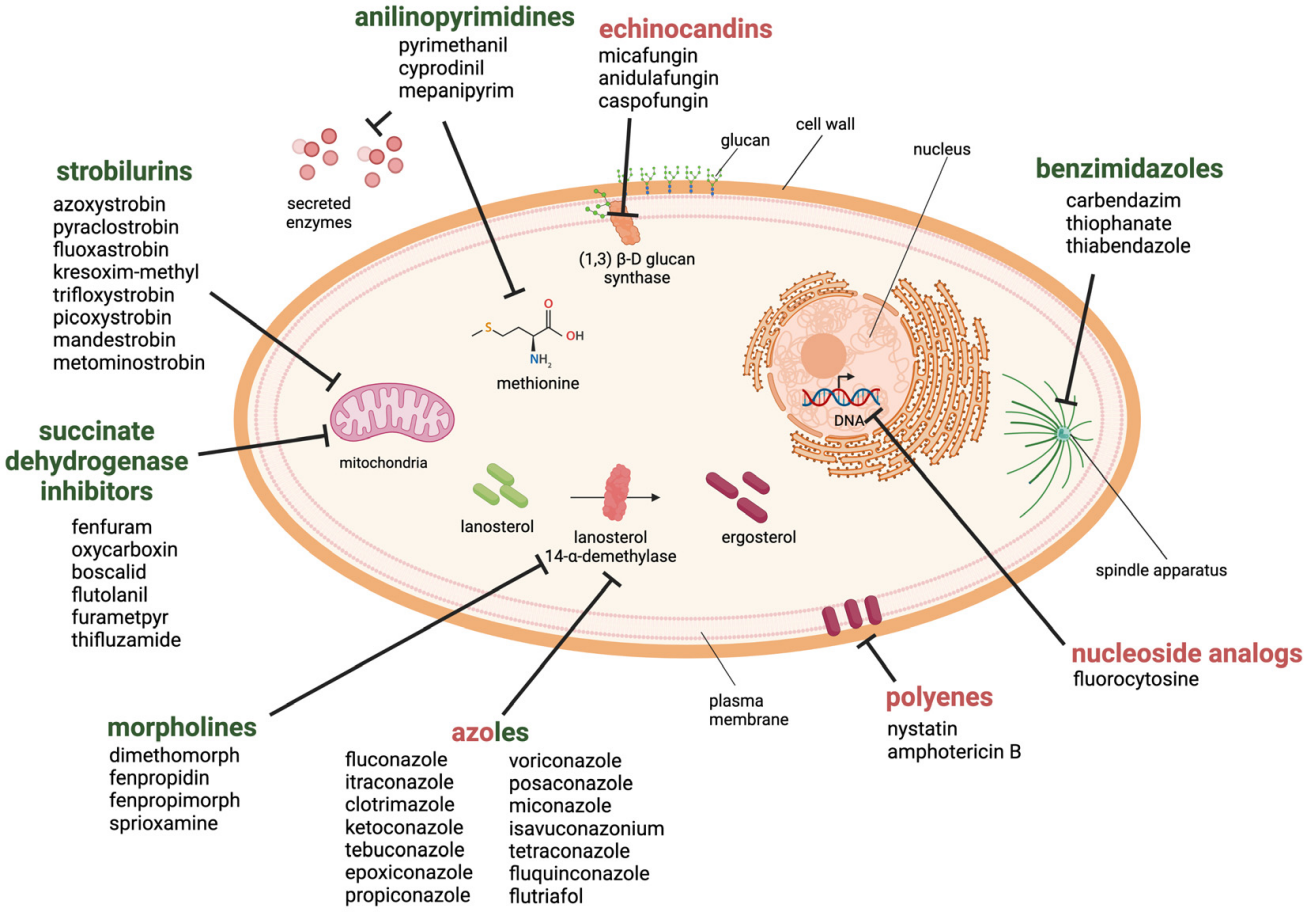
Antifungal drug classes used against fungal pathogens in the health and agricultural sector. The six classes of antifungal agents used in the agricultural sector are morpholines, anilinopyrimidines, strobilurins, succinate dehydrogenase inhibitors, benzimidazoles and azoles (green). Examples for clinically approved antifungal classes are azoles, polyenes, echinocandins and nucleoside analogs (red). Created with BioRender.com.

Currently, there are six main antifungal classes that are effective against plant pathogenic fungi (Brauer et al., 2019) (**Figure 3**). Benzimidazoles are heterocyclic aromatic organic compounds that affect the fungal cell by binding to β-tubulin, forming tubulin-benzimidazole interactions and thus inhibiting the microtubule assembly during mitosis. However, resistance development was observed within a year of its introduction to the market (Vasava et al., 2020).

Morpholines, on the other hand, target the fungal cell by inhibiting the Δ14-reductase and Δ8/Δ7-isomerase. This hinders the synthesis of ergosterol, which is a part of the fungal membrane (Mercer, 1991). Morpholines are used as fungicides or as a protective glazing agent for coating or waxing fruits and vegetable to inhibit fungal growth and expand the shelf life of the products (An et al., 2020). This practice is still approved worldwide in agriculture and post-harvest storage. However, some countries have imposed limitations or even prohibited its use due to its toxicity to consumers (Health Canada, 2019; European Union Food Additives Database, 2024).

The class of strobilurins and succinate dehydrogenase inhibitors interfere with the electron transfer chain of mitochondrial respiration. Strobilurins bind to the quinone outside binding pocket of cytochrome b and block electron flow by inhibiting the complex III. Instead, inhibitors of succinate dehydrogenase (complex II) affect tricarboxylic acid cycle and disrupts the mitochondrial electron transport chain. The activity of both ultimately leads to reduction/inhibition of adenosine triphosphate (ATP) synthesis (Balba, 2007; Fisher et al., 2018; Luo and Ning, 2022).

Anilinopyrimidines are currently classified as methionine synthesis inhibitors and have been shown to inhibit the secretion of fungal hydrolytic enzymes; however, their main molecular target remains elusive (Leroux et al., 2002).

Azoles are widely used in agriculture, and are one of the four main drug classes structurally and functionally similar to those applied in the health sector. They inhibit the lanosterol 14-α-demethylase, a crucial enzyme in the ergosterol biosynthesis. Azoles are commonly used to prevent or treat fungal infections in crops, humans, and livestock. There are currently 31 licensed azoles for crop protection in the agricultural sector and five licensed azoles available for clinical treatment (Fisher et al., 2018).

Further antifungal drugs used for the clinical treatment of fungal infections are shown in **Figure 3** as well. Polyenes interact primarily with ergosterol and disrupt the integrity of the plasma membrane. The mode of action has been linked to the pore-forming ability; although recent studies suggest that other mechanisms may also be involved, such as reactive oxygen species (ROS) generation or membrane dysfunction by ergosterol sequestration (Carolus et al., 2020). The class of nucleoside analogs currently only includes one member, the pyrimidine analog 5-fluorocytosine, which is transported into the fungal cell where it is deaminated to the toxic compound 5-fluorouracil. It blocks the pyrimidine metabolism and consequently the DNA and RNA synthesis (Vermes et al., 2000).

Finally, the echinocandins target the 1,3-β-D-glucan synthase and inhibit the enzymatic step that links uridine diphosphate glucose moieties to form β-D-glucan. The inhibition of this enzymatic step destabilizes the fungal cell wall (Perlin, 2011).

It could be assumed that this arsenal of antifungal agents is sufficient to combat any fungal disease in plants, animals, and humans alike. However, the application of these approved antifungals has revealed several drawbacks, such as undesirable side effects, which directly affect the well-being of the patients. Furthermore, the ever-growing worldwide population demands a high quantity of food, resulting in the development of crops for maximum yield in monocultures, making them susceptible to fungal pathogens. Medical advances allow to sustain especially vulnerable patient risk groups. This progress has led to the misuse and over-prescription of antifungal agents that may have a fungistatic rather than fungicidal mode of action, which has facilitated the emergence of antifungal resistance in fungal pathogens in public health and agricultural settings. As azoles are used in both, fields and patients, cross-resistance is observed (Fisher et al., 2018). This narrows the options for medical therapy and the use of biopesticides and results in treatment failure and increased patient morbidity and mortality (Odds et al., 2003; Sau et al., 2003; Brüggemann et al., 2009; Lewis, 2011; Hamill, 2013; Gupta et al., 2018; Revie et al., 2018).

Resistance development in fungal pathogens has been documented for nearly all antifungal classes and is driven by three main mechanisms that take place in the fungal cell: (1) decrease of drug concentration, (2) drug target alterations and (3) metabolic bypasses (Sanglard, 2016; Hokken et al., 2019). These mechanisms lead to the emergence of pathogens that have acquired resistance due to prolonged exposure to antifungal drugs in agricultural or clinical settings. They can also be the cause of the intrinsic resistance in specific fungal species (Pfaller and Diekema, 2004; Ribas e Ribas et al., 2016). Resistance mechanisms in pathogenic fungi are not only limited to a single drug class, but can contribute to the resistance against two or more antifungal classes, which is known as multi-drug resistance (Walsh et al., 2004; Healey et al., 2016; Chang and Heitman, 2019; Carolus et al., 2021; Logan et al., 2022). The failure of antifungal therapy increases the demand for new next-generation antifungals.

## 3 Next-generation antifungals

Two main strategies are being pursued in the search for new or improved antifungal agents: Firstly, to improve the efficacy of approved antifungals, they can be (i) modified structurally, (ii) combined with novel antifungal compounds to exploit synergistic effects and lower effective drug concentrations, or (iii) repurposed from approved drugs. The second approach is to screen and identify novel biomolecules and synthetic compounds with antifungal activity (Ngo et al., 2016).

After more than two decades of research, the FDA has recently approved the new antifungal drugs ibrexafungerp (formerly known as MK-3118 and SCY-078) and the tetrazole oteseconazole (VT-1161) for the treatment of vulvovaginal candidiasis (McCarthy, 2022; Lanier and Melton, 2024).

Several potential new compound classes are currently undergoing different stages of clinical development. For example, rezafungin (CD101), a novel echinocandin that inhibits 1,3-β-D-glucan synthase, is in clinical trial phase 3. For new antifungal classes with novel mechanisms of action, compounds such as olorofim (F901318) that inhibits dihydroorotate dehydrogenase (phase 2) or the pro-drug fosmanogepix (APX001) that targets the fungal enzyme Gwt1 and inhibits an early step in glycosylphosphatidylinositol (GPI)-anchor biosynthesis (phase 2) have been developed (Gintjee et al., 2020; Logan et al., 2022).

### 3.1 Small antimicrobial proteins and peptides

Antimicrobial proteins and peptides (AMPs) with low molecular mass (<100 amino acids) are a promising group of new antifungal molecules. These biomolecules are mostly cationic and cysteine-rich proteins and peptides that are amphipathic and produced by prokaryotic and eukaryotic organisms. In 2024, more than 3,900 putative AMPs have been annotated in the Antimicrobial Peptide Database (ADP3) (Wang et al., 2016). Most of these AMPs are a component of the innate immune system in higher eukaryotes. They exhibit broad antimicrobial activity against viruses, Gram-positive and Gram-negative bacteria, fungi, unicellular protozoa, and even cancer cells (Zasloff, 2002). In order not to go beyond the scope of this article, we would like to refer readers who are interested in AMPs from higher eukaryotes to other excellent reviews in this field (Wiesner and Vilcinskas, 2010; van Dijk et al., 2023).

However, both prokaryotic and eukaryotic microorganisms, which lack a canonical immune system, also produce AMPs, which may function as sensing molecules, supporting a primitive communication between the microbial cells and aiding in survival under competitive nutrient conditions in their respective ecological niches (Hegedüs and Marx, 2013; Meyer and Jung, 2018).

### 3.2 AMPs from Eurotiales with antifungal activity

Filamentous fungi are a rich source of AMPs and the genomes of fungi belonging to the order Eurotiales contain genes coding for AMPs (Sonderegger et al., 2018). Previous studies have demonstrated that these AMPs exhibit predominant, if not exclusive, antifungal activity, which is the basis for their designation as antifungal proteins (AFPs). The first studies on AFPs were conducted around 30 years ago and involved the *Aspergillus giganteus* antifungal protein AFP and the *P. chrysogenum* antifungal protein PAF (Wnendt et al., 1994; Marx et al., 1995). To avoid confusion with the previously defined abbreviation "AFP", which stands for antifungal proteins, we refer in the following to the *A. giganteus* antifungal protein as AgAFP.

#### 3.2.1 The structural characteristics of AFPs and their implications for their antifungal function

The cationic and cysteine-rich AFPs are small proteins consisting of 50-60 amino acids after cleavage of an N-terminal prepro-sequence that drives protein secretion and maturation. The pro-sequence is believed to protect the producing fungal cell from premature antifungal activity and is removed before or during protein release into the environment (Marx, 2004). No other post-translational modifications have been identified. A characteristic feature of AFPs is a high number of positively charged arginines, lysines and histidines which contributes to the peptides' overall positive charge and the presence of 6-8 conserved cysteines that form 3-4 disulfide bonds (Galgóczy et al., 2019).

A homology analysis of putative AFPs from Eurotiomycetes listed in MycoCosm portal (Grigoriev et al., 2014) distinguished four different protein clades: the *P. chrysogenum* antifungal protein (PAF) clade, the *A. giganteus* antifungal protein (AFP) clade, the bubble protein (BP) clade and the *Neosartorya fischeri* antifungal protein 2 (NFAP2) clade (Sonderegger et al., 2018). **Table 1** shows selected representatives of these clades, which have been the most extensively studied, such as AgAFP (Wnendt et al., 1994), PAF (Marx et al., 1995), *P. chrysogenum* antifungal protein B (PAFB) (Huber et al., 2018) and *P. chrysogenum* antifungal protein C (PAFC) (Holzknecht et al., 2020), *N. fischeri* antifungal protein (NFAP) (Kovács et al., 2011) and *N. fischeri* antifungal protein 2 (NFAP2) (Tóth et al., 2016), *Aspergillus niger* antifungal protein (AnAfp) (Lee et al., 1999), *Penicillium brevicompactum* bubble protein (BP) (Seibold et al., 2011), *Penicillium digitatum* antifungal protein B (AfpB) (Garrigues et al., 2017a, b), *Penicillium expansum* antifungal protein A (PeAfpA), *P. expansum* antifungal protein B (PeAfpB) and *P. expansum* antifungal protein C (PeAfpC) (Garrigues et al., 2018). Fungi can produce several AFPs that belong to different clades. For example, the genomes of *P. chrysogenum* and *P. expansum* contain three genes encoding AFPs that belong to distinct clades: in *P. chrysogenum* PAF, PAFB (PAF clade), and PAFC (BP clade) and in *P. expansum* PeAfp, PeAfpB (PAF clade) and PeAfpC (BP clade), respectively. *N. fischeri* genes code for two previously characterized AFPs, NFAP (PAF clade) and NFAP2 (NFAP2 clade), while one putative protein, NFBP (BP clade) awaits further investigation (Sonderegger et al., 2018).

**Table 1:** A selection of representative AFPs from Eurotiales and their physicochemical properties^&^.

clade^*^	AFP	producing organism	no aa/MW^$^	pI	Lys/Arg/His	reference
AFP	AgAFP	*Aspergillus giganteus*	51/5.8	9.3	12/1/0	Wnendt et al. (1994)
BP BP BP	BP PAFC PeAfpC	*Penicillium brevicompactum* *Penicillium chrysogenum* *Penicillium expansum*	64/6.6 64/6.6 64/6.7	7.7 7.7 6.9	4/4/1 2/6/1 3/5/1	Seibold et al. (2011) Holzknecht et al. (2020) Garrigues et al. (2018)
NFAP2	NFAP2	*Neosartorya fischeri*	52/5.6	9.0	7/0/2	Tóth et al. (2016)
PAF PAF PAF PAF PAF PAF PAF	AnAfp NFAP PAF PAFB PeAfpA PeAfpA PeAfpA	*Aspergillus niger* *Neosartorya fischeri* *Penicillium chrysogenum* *Penicillium chrysogenum* *Penicillium expansum* *Penicillium expansum* *Penicillium digitatum*	58/6.5 57/6.6 55/6.3 58/6.5 57/6.6 58/6.6 58/6.6	6.6 8.9 8.9 8.8 9.5 8.3 9.1	5/2/6 11/2/1 13/0/0 8/2/6 12/3/0 9/2/6 5/5/5	Lee et al. (1999) Kovács et al. (2011) Marx et al. (1995) Huber et al. (2018) Garrigues et al. (2018) Garrigues et al. (2018) Garrigues et al. (2017a, 2017b)

^&^Physicochemical properties were calculated by protparam (www.protparam.org). ^*^AFPs are listed according to their classification by clades which are defined based on the phylogenetic tree described in Sonderegger et al. (2018). **^$^** Molecular weight of the mature protein is given in kDa.

**Figure 4:**
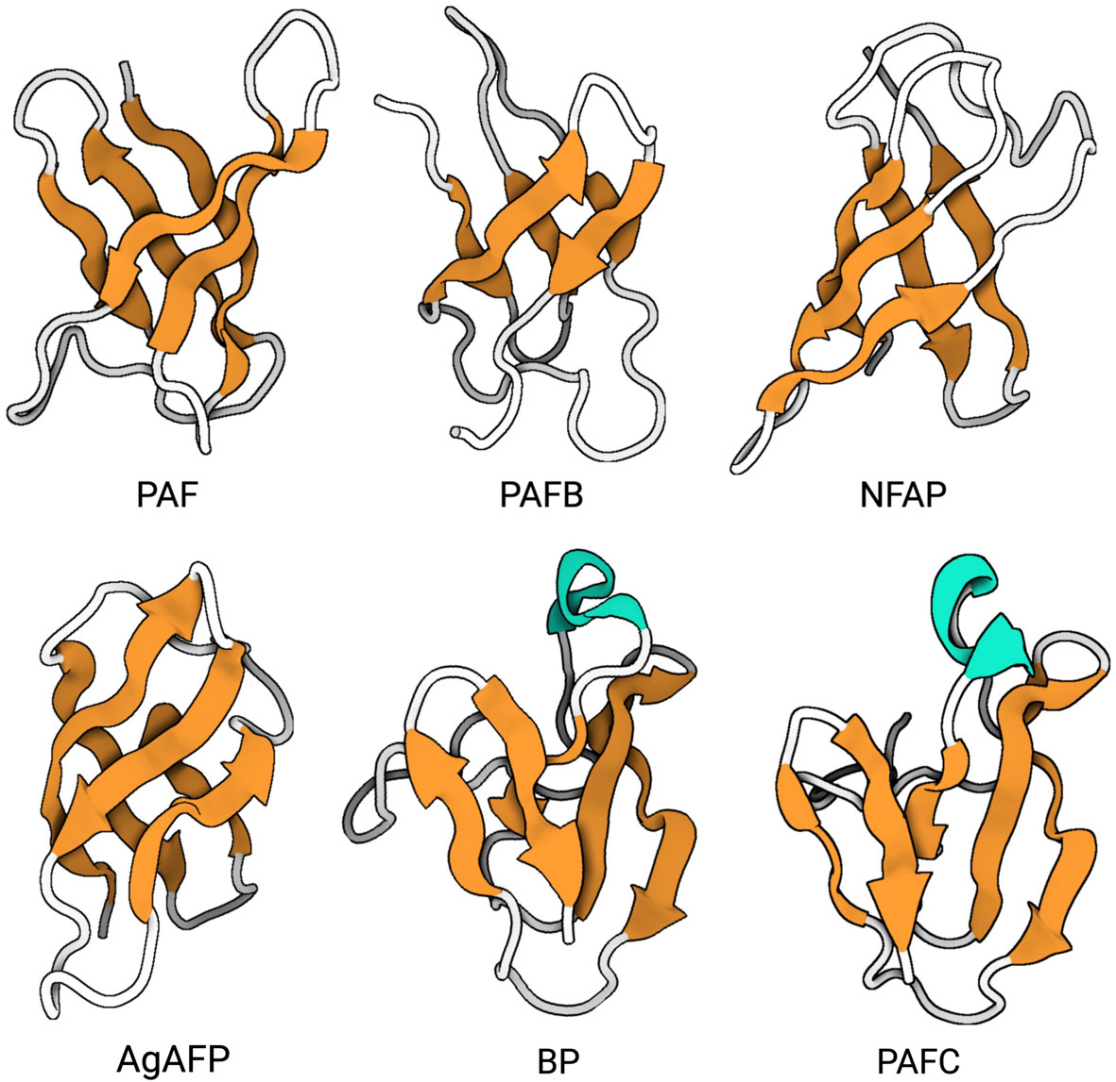
Representativ AFPs of fungal origin. Exchange (PDB: 6HAJ) for (PDB: 2MHV). AFP solution structures of selected AFPs, representing three of the four AFP clades from Eurotiales: The PAF clade, including PAF (PDB: 2MHV), PAFB (PDB: 2NC2) and NFAP (PDB: 5OQS), the AFP clade, including AgAFP (PDB: 1AFP), and the BP clade, including BP (PDB: 1UOY) and PAFC (PDB: 6TRM). The structure of representatives of the NFAP2 clade has not been characterized yet. Created with BioRender.com.

For the AFPs of *A. giganteus*, *N. fischeri* and *P. chrysogenum* the tertiary structure has been solved by NMR (Campos-Olivas et al., 1995; Batta et al., 2009; Galgóczy et al., 2017; Huber et al., 2018; Hajdu et al., 2019; Czajlik et al., 2021; Váradi et al., 2023b). Generally, most studied AFPs from Eurotiales possess five antiparallel β-strands connected by four loops. The β-strands fold into two antiparallel β-sheets that form a more or less rigid β-barrel structure (**Figure 4**). This is stabilized by 3-4 disulfide bonds between 6-8 conserved cysteines, whose bonding pattern could be predicted by computational approaches and proven by chemical synthesis of PAF and AgAFP (Váradi et al., 2013; Váradi et al., 2023a). The S-S bond pattern "abcabc" between the six cysteines present in NFAP (5oqs), PAF (2mhv), and PAFB (2nc2), "abbcac" in NFAP2 (8rp9) or "abcdabcd" and "abcabdcd" between the eight cysteines present in the AgAFP (1afp) and PAFC (6trm), respectively, is very important for the correct folding, high stability against harsh environmental conditions, such as high temperature, extreme pH, proteolytic degradation, or antifungal function (Batta et al., 2009; Váradi et al., 2013; Galgóczy et al., 2017; Váradi et al., 2023a).

The three-dimensional (3D) NMR structure data of PAF and PAFB were supported by results obtained with X-ray crystallography (Alex et al., 2019; Guagnini et al., 2021). However, unlike the published structures of the other AFPs, PAFC has an extended slightly less ordered N-terminal region with a short helix-like conformation, shorter β-strands and lacks the typical β-barrel structure. Instead, it has a positively charged cavity and a hydrophobic core that is surrounded by the four disulfide bonds (Czajlik et al., 2021). The NMR-based 3D structure of PAFC closely resembles the X-ray crystal structure of the antifungal BP (1uoy) of *P. brevicompactum*, with which PAFC shares approx. 80% sequence identity (Olsen et al., 2004; Czajlik et al., 2021).

Approaches using chemical synthesis and amino acid exchanges in the AFPs helped to identify distinct motifs that might play a role in their correct folding and antimicrobial function (Batta et al., 2009; Garrigues et al., 2017a; Sonderegger et al., 2017; Fizil et al., 2018; Sonderegger et al., 2018; Váradi et al., 2023a; Váradi et al., 2023b).

One interesting structural feature common to all AFPs is the highly conserved consensus sequence GXCX_3-9_C (X being any amino acid), which is also commonly found in disulfide-stabilized AMPs produced by organisms from all kingdoms. The motif consists of two antiparallel β-strands connected by a short turn region, which folds into a structure resembling the Greek letter gamma “γ” (γ-core motif) (Yount and Yeaman, 2006). Previous studies have suggested that the γ-core motif plays a role in supporting the specific interaction of AMPs with membrane targets (Yount and Yeaman 2006; Yeaman et al., 2007; Yount et al., 2007; Utesch et al., 2018).

**Figure 5:**
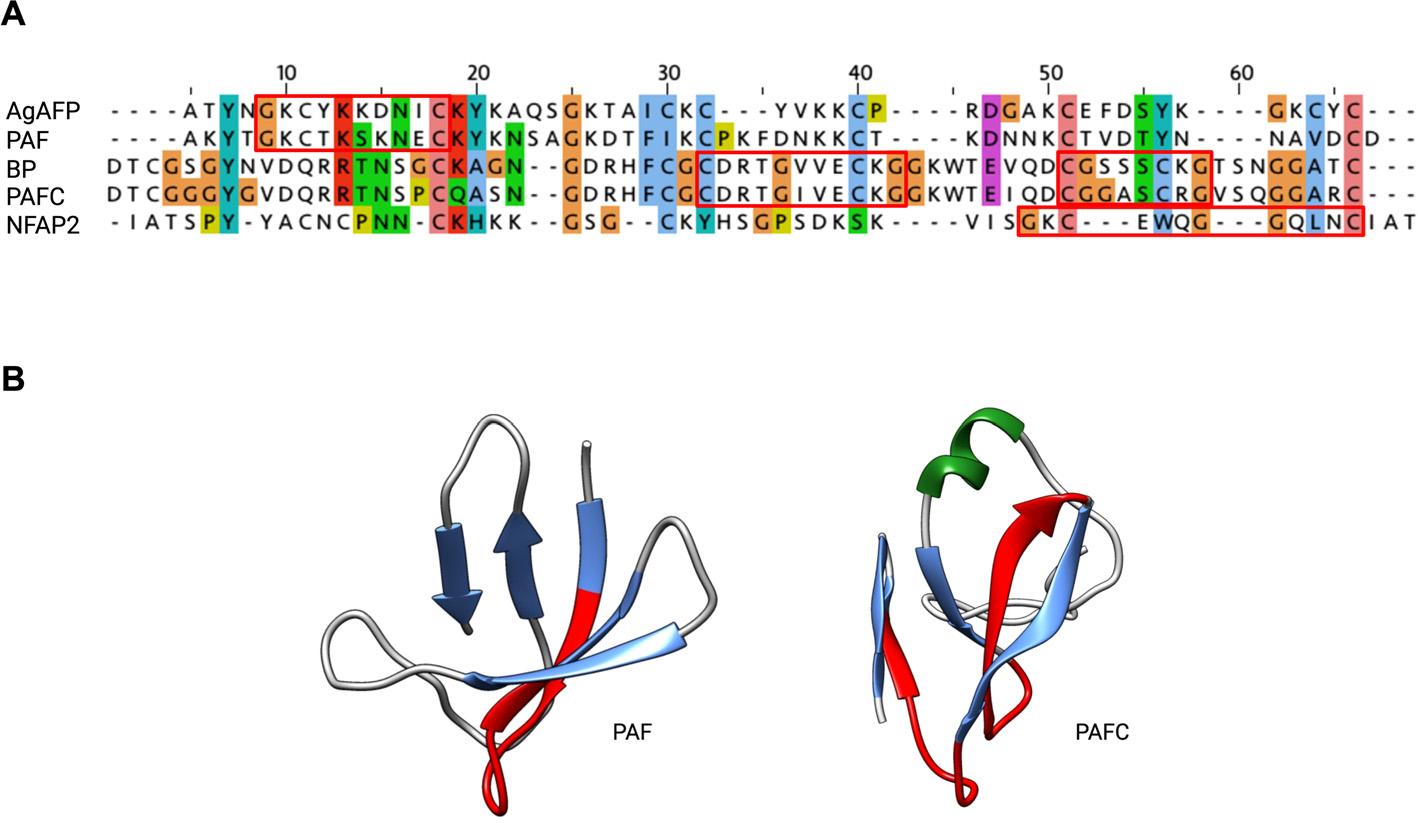
The evolutionary conserved γ-core motif. The γ-core motif (framed in red) consists of the consensus sequence GXCX_3-9_C, which can be present in the **(A)** dextromeric isoform (X_1-3-_GXC-X_3-9-_C), found in AgAFP, PAF and NFAP2 or in the levomeric isoforms (C-X_3-9-_CXG-X_1-3_), found in BP and PAFC. This motif is also structurally conserved, as it **(B)** forms the Greek letter “γ” by two antiparallel β-strands, which are connected by an interposed short turn region, shown in red in PAF (PDB: 2MHV) and PAFC (PDB: 6TRM). JALVIEW with ClustalW alignment and clustalX default colour scheme, protein structures were visualized with Chimera (Pettersen et al., 2004), α-helix is depicted in green, β-strands in blue. Created with BioRender.com.

Phylogenetic analyses revealed conserved positions of the γ-core motif in each AFP clade. Members of the PAF-, AFP-, and NFAP2-clade contain a dextromeric γ-core motif (X_1-3_-GXC-X_3-9_-C) in their sequence, whereas members of the BP-clade exhibit two γ-core motifs in the levomeric form (C-X_3-9_-CXG-X_1-3_; **Figure 5**). These motifs can have a positive, negative, or neutral charge, which may affect their antifungal activity and mode of action (Sonderegger et al., 2018). The role of the γ-core motif in AFPs, however, is a topic of debate. A recent study that used computational mining of protein databases to predict structure-activity did not support the idea that the γ-core motif is a unifying structural signature of potential AFPs (Feurstein et al., 2022). While Sonderegger et al. (2018) has demonstrated the antifungal activity of the synthetic peptide that spans the γ-core motif of PAF (Pγ), no or only moderate activity of synthetic peptides representing this motif from other AFPs, like the *P. chrysogenum* PAFB, the *P. digitatum* PdAfpB or the *N. fischeri* NFAP and NFAP2, has been detected under the experimental conditions applied, possibly due to lower net charge and lower hydrophilicity of this specific motif (Garrigues et al., 2017a; Tóth et al., 2018; Huber et al., 2020). The γ-core peptides can also have species-specific activity, like the peptide PCγ17, which spans the levomeric γ-core of PAFC. This peptide effectively inhibits the growth of mold model *Neurospora crassa*, but not that of the opportunistic human pathogenic yeast *C. albicans* (Czajlik et al., 2021).

Apart from its potential role in antifungal activity, the γ-core motif is also discussed as a structural determinant for the characteristic folding pattern of AFPs (Galgóczy et al., 2017; Hajdu et al., 2019; Váradi et al., 2023a; Váradi et al., 2023b).

However, those γ-core peptides with antifungal activity are interesting molecules to investigate their mechanism of action and their potential to be developed into effective peptide-based antifungal drugs through rational design. The peptide Pγ was synthesized according to the N-terminally located dextromeric γ-core motif of PAF and exhibited antifungal activity, although it was less effective than the full-length protein. This suggests that additional structural elements present in the full-length protein are required to exert full activity. To enhance the antifungal activity, specific amino acids in Pγ were exchanged, creating the peptide variant Pγ^opt^ with an elevated net charge and higher hydrophilicity which resulted in increased antifungal activity. The attempt to optimize the γ-core in the full-length PAF by exchanging the same amino acids as in Pγ^opt^ increased the structural flexibility of PAF^opt^ and significantly improved anti-*Candida* efficacy (Sonderegger et al., 2018). A similar approach was initiated to improve the antifungal efficacy of the γ-core peptides of NFAP and NFAP2 by rational design. The increase in the positive net charge of the peptide γ^NFAP^ resulted in improved antifungal activity of the designed peptides γ^NFAP^-opt and γ^NFAP^-optGZ, irrespective of their hydrophilic/hydrophobic character (Tóth et al., 2020b). Similarly, the attempt to improve the antifungal efficacy of the peptide γ^NFAP2^ by modulating its physicochemical properties by amino acid exchange resulted in a more effective peptide γ^NFAP2^-opt (Tóth et al., 2022).

Other attempts were performed to ensure structural integrity and improve antifungal efficacy by cysteine-serine substitutions and S-tert-butylation of cysteine residues. These studies demonstrated that S-tert-butyl-protected cysteines help to maintain the structural integrity and broaden the antifungal spectrum; however, no improvement of the antifungal efficacy was achieved (Váradi et al., 2024).

#### 3.2.2 Antimicrobial spectrum

In this chapter, we list numerous fungal species that have been reported to be sensitive to AFPs, including animal/human pathogenic fungi, phytopathogens, and fungi widely used as model organisms in life science research. Their biology, taxonomy, occurrence and distribution, and importance in research and as pathogens in medicine, and agriculture will not be discussed here. Without claiming to be exhaustive, we would like to refer the reader to some excellent articles dealing with these aspects (Brown et al., 2012; Silva et al., 2012; Kwon-Chung et al., 2014; Perez-Nadales et al., 2014; Nobile and Johnson, 2015; Chowdhary et al., 2017; Doehlemann et al., 2017; Rajasingham et al., 2017; Denning et al., 2018; Petrucelli et al., 2020; Peng et al., 2021; Carpouron et al., 2022; Corbu et al., 2023; Gómez-Gaviria et al., 2023; Pham et al., 2023; Stemler et al., 2023; Dellière et al., 2024; Hill et al., 2024; Morrissey et al., 2024).

**Figure 6:**
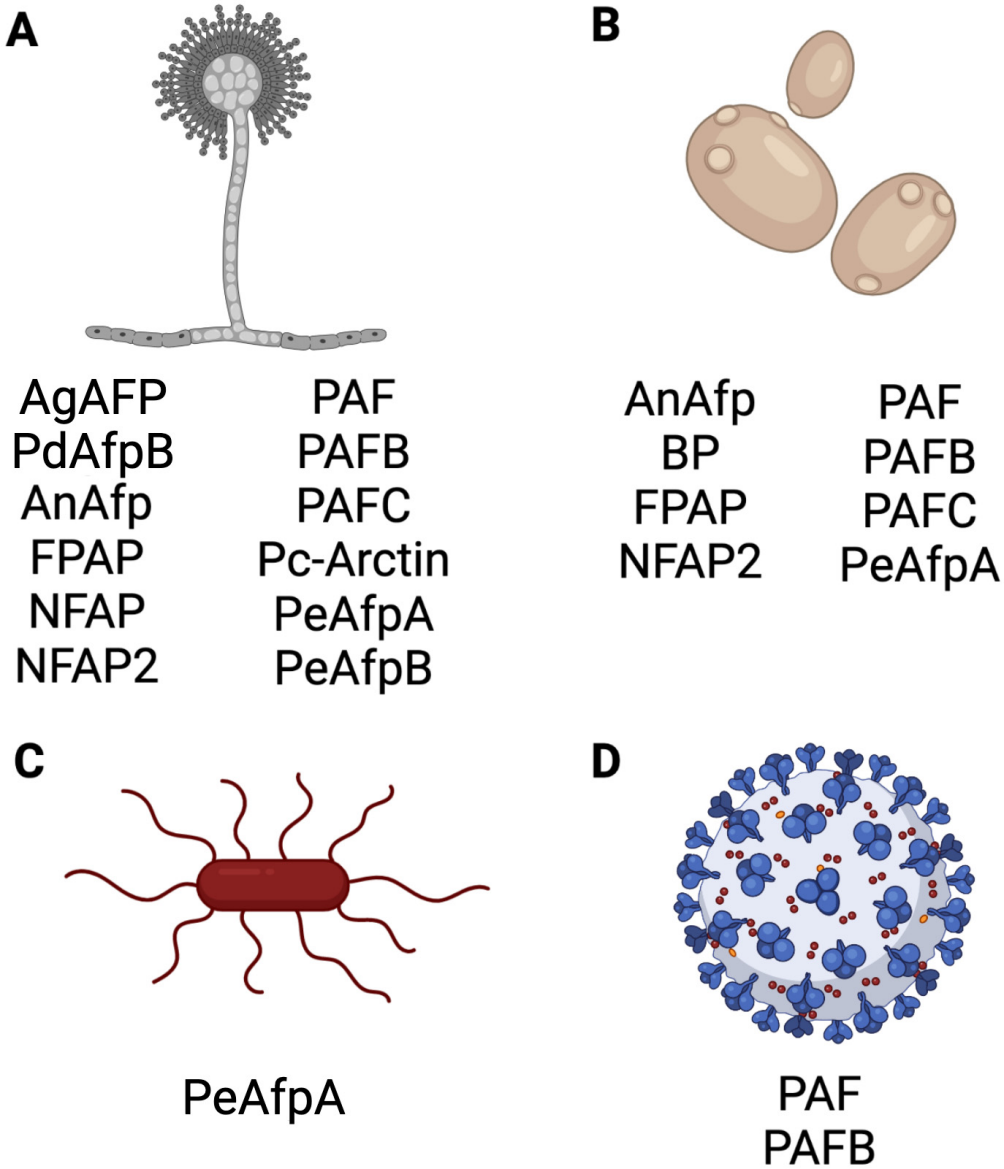
Antimicrobial activity of selected AFPs of Eurotiales. Activity of representative AFPs against **(A)** filamentous fungi, **(B)** yeasts, **(C)** bacteria and **(D)** virus. Created with BioRender.com.

The Eurotiales AFPs demonstrate antimicrobial activity against a wide range of different fungi (**Figure 6**). Studies have shown growth inhibition of various filamentous fungal species, including opportunistic human pathogens such as *Aspergillus flavus*, *Aspergillus fumigatus, Aspergillus nidulans, Aspergillus niger*, *Aspergillus terreus*, *Paecilomyces variotii*, and the dermatophytes *Microsporum canis*, *Microsporum gypseum*, *Trichophyton rubrum*, *Trichophyton mentagrophytes* and *Trichophyton tonsurans*.

Activity against the model fungi *Alternaria longipes*, *Aspergillus nidulans*, *Neurospora crassa* and *P. chrysogenum*, and plant pathogenic fungi, such as *Arthroderma vanbreuseghemii*, *Blumeria graminis*, *Botrytis cinerea*, *Fusarium oxysporum*, *Gibberella moniliformis*, *Magnaporthe oryzae*, *P. expansum, P. digitatum, Penicillium italicum, Phytophthora infestans*, *Puccinia recondita*, *Sclerospora graminicola* and *Trichoderma viride* has also been documented. Additionally, activity against the opportunistic human pathogenic yeasts *C. albicans, Candida glabrata, Candida guillermondii, Candida krusei, Candida lusitaniae, Candida parapsilosis, Candida tropicalis* and *C. auris*, and the model yeasts *Saccharomyces cerevisiae* and *Schizosaccharomyces pombe* were observed (Vila et al., 2001; Marx, 2004; Girgi et al., 2006; Barna et al., 2008; Galgóczy et al., 2008; Marx et al., 2008; Meyer, 2008; Seibold et al., 2011; Chen et al., 2013; Tóth et al., 2016; Huber et al., 2018; Holzknecht et al., 2020; Huber et al., 2020; Czajlik et al., 2021; Gandía et al., 2021; Kovács et al., 2021; Manzanares et al., 2024).

While most AFPs revealed no antibacterial activity, only the PeAfpA from *P. expansum* was found to inhibit the proliferation of *Agrobacterium tumefaciens*, *Bacillus subtilis* and *Escherichia coli* (Gandía et al., 2020).

Finally, few AFP examples exist for which antiviral activity has been reported, like the *P. chrysogenum* PAF and PAFB (Huber et al., 2018).

#### 3.2.3 Mode of action

In general, positively charged, amphipathic AMPs are suggested to have two modes of action: (i) direct binding to the outer cell layers (cell wall and/or membrane), following cell lysis or (ii) uptake of the AMPs into the sensitive target cell without membrane disruption and inhibition of essential cellular functions by binding to intracellular targets, such as proteins, lipids or nucleic acids (Sani and Separovic, 2016; Le et al., 2017). However, several studies in search for possible interaction molecules suggested that growth inhibitory mechanisms might differ between AMPs, and targets still remain elusive. The same accounts for AFPs from Eurotiales.

The fungal cell wall serves as the initial barrier for AFPs. Its main components are mannoproteins, α- and β-glucans, and chitin. Several approaches aimed at investigating the role of the fungal cell wall, and specifically, chitin as a potential target of AFPs. Hagen et al. (2007) demonstrated that the AgAFP has the ability to bind chitin. Similarly, the *P. digitatum* AfpB is assumed to interact with the cell wall chitin before reaching the fungal cell membrane (Bugeda et al., 2020). However, oomycetes, such as *P. infestans* and *S. graminicola*, are sensitive to the growth inhibitory activity of AgAFP despite the absence of chitin in their cell wall (Vila et al., 2001; Girgi et al., 2006). Moreover, Batta et al. (2009) found no evidence to suggest that PAF from *P. chrysogenum* interacts with chitin.

Other studies have demonstrated that AFPs are capable of crossing the cell wall and translocating the fungal cell membrane in a time- and concentration-dependent manner. This suggests that intracellular molecules may be targeted (Martinez Del Pozo et al., 2002; Moreno et al., 2006; Martín-Urdiroz et al., 2009; Virágh et al., 2015; Huber et al., 2019b, 2020). However, defined targets have remained elusive.

It is assumed that an electrostatic interaction occurs between the positively charged AFPs and the negatively charged components of the cell membrane, e.g. phospholipids. This may be further supported by hydrophobic interactions between the amphipathic domains of the AFP and the cell membrane. Studies have demonstrated that PAF protein variants with a lower net charge exhibit reduced antifungal activity. Alterations in the surface composition that affect charged, aromatic, and hydrophobic patches of PAF, resulted in a loss or attenuation of antifungal activity, even when the overall structure was maintained (Batta et al., 2009; Sonderegger et al., 2016, 2017). The interaction of PAF and PAFB with the negatively charged fungal cell membrane is based on their positive net charge. It was further shown that the initially suggested membrane lipid components, glucosylceramides, are not their direct targets of the sensitive fungus *N. crassa*. In contrast, a recent study using model fungi like *S. cerevisiae*, *A. niger*, and *Pichia pastoris* has indicated that fungal glucosylceramides may represent potential direct targets or may facilitate the binding of AgAFP and the interaction may occur via the γ-core motif of AgAFP (Utesch et al., 2018; Paege et al., 2019).

It is crucial to note that the binding of AFPs to the fungal cell membrane alone may not be sufficient to induce cell death. In order to have an antifungal effect, a complex multi-step mechanism must take place including the uptake of the AFP into the fungal cell. This excludes several AFPs from being considered canonical membrane-disrupting molecules. The uptake of PAF, PAFB, and PAFC was observed only in germinating conidia or hyphae, but not in conidia, and could be blocked by reducing the cell metabolism. This indicated that these AFPs are internalized into the fungal cell by an endocytosis mechanism (Huber et al., 2019b; Holzknecht et al., 2020; Huber et al., 2020). In contrast, NFAP was found to be passively internalized in *A. nidulans*, resulting in subsequent cell death (Virágh et al., 2015). Moreover, PAF was found to induce the hyperpolarization of the fungal cell membrane at hyphal tips, cause the efflux of ions into the supernatant, elevate the cytoplasmic Ca^2+^ levels and induce intracellular ROS (iROS). These effects were accompanied by DNA strand breaks, and mitochondrial disintegration, all of which are known to be markers for apoptotic cell death (Kaiserer et al., 2003; Leiter et al., 2005; Binder et al., 2010, 2015; Sonderegger et al., 2017; Huber et al., 2019b; Holzknecht et al., 2020; Huber et al., 2020).

Several reports describe that when AFPs interact with the fungal cells, they may activate signaling pathways. AgAFP, the antifungal protein AFP_NN5353_ from *A. giganteus* strain MDH 18894 and AnAFP induce the cell wall integrity (CWI) signaling pathway, which increases α-1,3-glucan synthase activity and the fortification of the cell wall. This indicates that the fungal cell experiences cell wall stress when exposed to these AFPs (Hagen et al., 2007; Binder et al., 2011; Meyer and Jung, 2018). The protein-host interaction of the *P. digitatum* AfpB can be divided into three stages: (i) interaction with the cell wall, (ii) energy-dependent uptake into the fungal cell, and (iii) induction of iROS, G-protein signaling, MAPK signaling, and subsequent cell death (Bugeda et al., 2020). The *P. chrysogenum* PAF and *N. fischeri* NFAP have been demonstrated to interfere with the heterotrimeric G-protein and the cyclic adenosine monophosphate (cAMP)/protein kinase A signaling pathway (Leiter et al., 2005; Virágh et al., 2015). Similarly, the disruption of the calcium homeostasis has been shown in *A. niger* in response to the treatment with AFP_NN5353_ (Binder et al., 2011).

The antifungal mode of action has been predominantly studied in filamentous fungi. However, recent studies have attempted to elucidate the mechanism of AFPs in the baker’s yeast *S. cerevisiae* and the opportunistic human pathogenic yeast *Candida* spp. A multidisciplinary approach was employed to investigate the mode of action of the PeAfpA from *P. expansum* on *S. cerevisiae*. It was found that PeAfpA interacts with the cell wall before being internalized. Uptake can occur through energy-dependent or independent mechanisms. In the response to PeAfpA treatment, *S. cerevisiae* activated the CWI pathway and cAMP-PKA signaling. Furthermore, phosphatidylinositol metabolism and the RNA polymerase II mediator complex ROX3 were reported to play a role in the yeast's defense strategy (Giner-Llorca et al., 2023) NFAP2, PAF, PAFB and PAFC were found to be effective against a range of *Candida* species, including drug-resistant isolates (Tóth et al., 2016; Sonderegger et al., 2018; Holzknecht et al., 2020; Huber et al., 2020). In contrast to the other AFPs, NFAP2 demonstrated activity even in high cationic strength medium. It disrupts the plasma membrane of sessile cells growing in a biofilm, and is effective against fluconazole-resistant *C. albicans* and multi-drug resistant *C. auris* isolates (Tóth et al., 2016; Kovács et al., 2019, 2021). Following their uptake into *C. albicans* by an energy-dependent mechanism, PAF, PAFB, and PAFC initially localize in vacuoles before being released into the cytoplasm, where they induce iROS and ultimately cell death (Sonderegger et al., 2018; Holzknecht et al., 2020; Huber et al., 2020). These data collected with yeast cells suggest a mode of action that is similar to that observed with filamentous fungi.

## 4 First steps toward an antifungal application of Eurotiales AFPs

To be considered for future use as antifungal therapeutics, AFPs must meet a number of requirements. These include safety and efficacy, which can initially be tested in a small laboratory setting. Subsequently, larger scale studies can be conducted to address issues such as pharmacokinetics, formulation, and bioavailability. The first steps to test the applicability of Eurotiales AFPs have been successfully taken, including testing their tolerability and curative potential in mammalian cell culture and using 3D reconstructed human tissue, plant, and animal models.

### 4.1 Antifungal efficacy against opportunistic human pathogens

*In vitro* studies conducted in mammalian cell culture revealed that no cytotoxic effects were induced by fungal AFPs. For example, PAF, its γ-core variants and γ-core peptides, and NFAP2 were tested on primary human skin cells (Sonderegger et al., 2018; Kovács et al., 2019), PAF on human monocytes, white blood cells, umbilical vein endothelial cells and colonic epithelial cells, and on rat neuronal cells and astrocytes, and skeletal muscle fibers (Szappanos et al., 2005; Tóth et al., 2020a), PAFB on human epithelial cells (Huber et al., 2018), and AgAFP on human umbilical vein endothelial cells and whole blood cells, on mouse skeletal muscle fibers, and rat astrocytes and neuronal cells (Szappanos et al., 2006).

*In vivo* efficacy studies have been conducted to demonstrate the safety and antifungal activity of PAF and NFAP2 against *A. fumigatus* and *C. albican*s, respectively, using mouse models. Neither PAF nor NFAP2 application in mice had any adverse effects (Palicz et al., 2013; Kovács et al., 2019). The intranasal treatment of a pulmonary *A. fumigatus* infection in mice with PAF did not cure but delayed their mortality (Palicz et al., 2013). Furthermore, the intraperitoneal administration of PAF in combination with amphotericin B significantly prolonged the life span of the infected animals (Palicz et al., 2016). Similarly, the treatment of a murine model of vulvovaginitis with NFAP2 reduced the cell number of fluconazole-resistant *C. albicans* cells, and when combined with fluconazole, an enhanced antifungal efficacy was observed (Kovács et al., 2019).

The antifungal efficacy against epidermal fungal infections was investigated using the PAF γ-core variant, PAF^opt^, as well as PAFB, PAFC and NFAP2 in an *in vitro* full-thickness (FT) 3D skin model infected with *C. albicans*. All applied AFPs were well tolerated by the FT skin model, which represents an epidermis and a dermis and shows similarity to the architecture, physiology and metabolic competence of normal human skin. The AFP treatment reduced the fungal load and penetration depth of *C. albicans* in the infected *in vitro* models. Additionally, the epidermal permeability barrier was restored and IL-8 secretion was reduced (Holzknecht et al., 2022).

A recently published study showed that NFAP and NFAP2 may reduce the risk of allergic reactions caused by mold spores that infest indoor masonry (Dán et al., 2024).

### 4.2 Efficacy of AFP in protecting plants from phytopathogen infection

AFPs were applied on plants or parts thereof to investigate their protective potential against infection with phytopathogens. For example the *A. giganteus* AgAFP inhibited infection with *M. grisea* in rice plants (Vila et al., 2001), *B. cinerea* in *Pelargonium×hortorum* (geranium) leaves (Moreno et al., 2003), *F. oxysporum* in tomato roots (Theis et al., 2005). In plant disease prevention experiments, the *P. chrysogenum* PAF demonstrated efficacy in slowing down the progression of the disease induced by *B. graminis* in barely and *P. recondita* in wheat, respectively (Barna et al., 2008). PAF, PAF^opt^, PAFC and its derived γ-core peptide Pγ^opt^ exhibited anti-fungal activity in reducing infection of tomato leaves by *B. cinerea* (Tóth et al., 2020a; Czajlik et al., 2021). The *N. fischeri* NFAP and its derived γ-core peptide γ^NFAP^-opt acted similarly and inhibited *B. cinerea* growth in tomato leaves (Tóth et al., 2022).

Alternative approaches have been tested in which transgenic plants were created that express the AgAFP encoding *afp* gene thus increasing resistance against fungal infection. The experiments revealed that the plant tissue could be at least partially protected from the infection and expansion of phytopathogens, for example *Erysiphe graminis* in wheat (Oldach et al., 2001), *Magnaporthe grisea* in rice (Coca et al., 2004; Moreno et al., 2006), *P. substriata* and *S. graminicola* in pearl millet (Girgi et al., 2006), or *Rosellinia necatrix* in olive plants (Narvaez et al., 2018). Improved resistance against *B. cinerea* infection in the transgenic tobacco (*Nicotiana benthamiana*) expressing AgAFP or PdAfpB were also reported (Shi et al., 2019).

A novel approach was initiated by evaluating the efficacy of a fusion protein comprising a *Fusarium*-specific antibody and AgAFP in protecting transgenic *Arabidopsis thaliana* and wheat from *Fusarium* infection and in preventing mycotoxin contamination of cereal grains. The study proved that the expression of the fungal-specific antibody-AFP fusion protein was more effective than the expression of the antibody or the AFP alone in the transgenic plants (Peschen et al., 2004; Hu et al., 2008; Li et al., 2008).

### 4.3 AFPs in food preservation

AgAFP was found to be highly effective in inhibiting the growth of *Fusarium* spp., the primary contaminant of raw barley, which is frequently utilized as the initial substrate in the malting process. The application of AgAFP during malt production at laboratory scale and in a pilot plant study demonstrated a higher inhibitory potential than other common disinfectants, including ozone, hydrogen peroxide, and chlorine dioxide. This was achieved without any adverse effects on the quality of malt and wort (Barakat et al., 2010).

Some species of the *Penicillium* genus that produce AFPs are simultaneously phytopathogens and cause severe post-harvest fruit diseases. The *P. expansum* PeAfpA was effective in controlling the experimental infection of oranges with *P. digitatum* and of apples with *P. expansum* (Garrigues et al., 2018; Gandía et al., 2020). Another study evaluated the potential of the AFPs of *P. chrysogenum*, PAF, PAFB, and PAFC, and of *N. fischeri*, NFAP2, to control *Penicillium* decay in comparison to PeAfpA. The results indicated that PAFB was the most effective, apart from PeAfpA, in delaying the infection of orange fruits with *P. digitatum* and *P. italicum*, and the infection of apple fruits with *P. expansum* (Gandía et al., 2021). NFAP and its derived γ-core peptides, γ^NFAP^-opt and γ^NFAP^-optGZ, were effective in preventing the decay of tomato fruits caused by *Cladosporium herbarum* infection (Tóth et al., 2020b).

## 5 Discussion

### 5.1 The potential of AFPs as a new antifungal strategy

The increasing prevalence of resistant fungal pathogens and the frequent failure of licensed antifungal drugs to provide effective treatment highlight the urgent need for the development of new antifungal agents. AFPs derived from fungal sources are a plentiful and promising class of bioproducts that may serve as the next generation of therapeutics.

AFPs exhibit significant benefits as potential future drugs. Being naturally occurring proteins, they are biodegradable and less likely to contaminate the environment in comparison to synthetic antifungals. Mechanisms of action different to those of standard antifungal drugs that are currently on the market, and a fungicidal mode of action may allow treatment options for infections with drug-resistant fungal pathogens and reduce the risk of fungi developing resistance. The combination of AFPs with licensed antifungal drugs has the potential to achieve a synergistic effect, and increasing both the antifungal spectrum and efficacy, while at the same time minimizing the risk of resistance development. This opens up the possibility for long-term disease management. Moreover, their selectivity for fungi promise low toxicity and good tolerability in humans, animals, and plants. Finally, the option to further modulate and improve the antifungal activity by rational design is supported by having gained insight into the structure-function relationship of AFPs.

However, these advantages are paired with challenges that need to be solved. Being proteins they may be potentially immunogenic and elicit adverse reactions upon administration. Furthermore, their protein-/peptide-nature may render them susceptible to proteolytic degradation by fungal- or host-specific enzymes, which demands the development of effective delivery systems to ensure stability and bioavailability *in vivo*. The manufacturing of protein-based drugs on a large scale may be complex and expensive. Advances in biotechnology, however, may help to reduce costs. In addition to chemical synthesis, recombinant expression systems using microorganisms as cell factories in combination with production in large-volume fermenter systems represent a promising avenue for the production of AFP-based drugs (Batta et al., 2009; López-García et al., 2010; Sonderegger et al., 2016; Váradi et al., 2018; Huber et al., 2019a).

One key question has remained unanswered, the identification of the fungal target of Eurotiales AFPs. To the best of our knowledge, no reports have evidenced the identification of a direct target molecule to which any of the AFPs under study bind in order to execute their antifungal activity. The knowledge of a cellular binding target would open new perspectives for the creation of AFP derivatives with improved antifungal efficacy by rational design and foster the development of AFP-based drugs.

### 5.2 Promising applications for AFPs

The data collected from the experiments described in the chapters 3.2.2 and 3.2.3 indicate that AFPs have potential for use in a wide range of applications. These can be as diverse as the properties and areas of application of AMPs (Ricardo et al., 2020; Souza, 2022; Răileanu et al., 2023; Tang et al., 2023). Because some AFPs are sensitive to high ionic strength environments, they often appear to be drastically impaired or even inactive in *in vitro* susceptibility testing, and are less suitable for systemic therapy in the veterinary or clinical settings. Instead, they may be more promising for topical treatment of fungal infections, affecting for example the skin, nails, the oral and vaginal mucosa. In addition, the activity of AFPs against fungi that are a source of allergens envisions the control of indoor fungal infestations.

Due to their broad-spectrum activity against plant pathogens, AFPs are also attracting attention in the field of crop protection and food preservation, where they are seen as natural and sustainable molecules that can regulate the growth of phytopathogenic and post-harvest fungi. AFPs could be applied to crops and plants to limit the spread of phytopathogens in the fields and prevent harvest loss. They may also be used in food preservation in order to effectively prevent food spoilage caused by the secondary growth of filamentous fungi during storage in an efficient manner.

Additionally, AFPs may be employed in the creation of coatings designed to impede fungal biofilm formation on medical devices, such as catheters, artificial dentures, implants, and surgical instruments, thus effectively preventing infections. The safeguarding and sustainable protection of objects, such as masonry, books, sculptures, paintings, and other materials, may represent another potential area of application that counteracts biodeterioration of human cultural heritage.

### 5.3 Challenges in moving AFPs from the laboratory to industry

Despite the considerable number of scientific research articles on AMPs that have been identified as potent drug candidates, a comparably small number of patents have been granted (Kosikowska and Lesner, 2016). Only few of the promising candidates have reached clinical trials, although there is an increasing demand for peptide-based drugs (Fernández de Ullivarri et al., 2020; Rodríguez-Castaño et al., 2023; Cresti et al., 2024). The AFPs originating from Eurotiales could meet this demand.

However, a discrepancy persists between academic and industrial realms, resulting in a gap in the translation of findings from basic research to the market launch of the product (Kampers et al., 2021). This can be attributed to a number of factors, including toxicity, instability, and low efficacy, which may result in the failure to further consider proteins and peptides for *in vivo* application. In the majority of instances, the efficacy data obtained from *in vitro* studies fails to accurately reflect the results observed in clinical or agricultural settings. Antimicrobial drug compounds are subjected to standard *in vitro* setups, employing techniques such as broth microdilution assays and agar diffusion tests, with the objective of determining fungi that are susceptible to the test compound and the minimal inhibitory concentration (MIC) at which pathogen growth is reduced or inhibited. This method does not allow for the drawing of any conclusions regarding the fungicidal or fungistatic mode of action, nor does it reflect the environmental conditions that would be present during the application of AFPs. This impairs the reliability of predictions regarding the *in vivo* outcome.

Caution must also be exercised when interpreting the species-specificity of AFPs determined in broth microdilution assays. This is due to the fact that these assays, even when performed under standardized CLSI or EUCAST conditions may vary between the different labs (CLSI, 2017; Arendrup et al., 2021). The protocols recommend the use of RPMI 1640 medium, a cell culture medium that is believed to closely resemble the extracellular fluid of the human body (Moore et al., 1967). However, it should be noted that RPMI 1640 differs from the *in vivo* situation in terms of oxygen, iron, pH, carbohydrate and salt concentration, which can influence the prediction of compound activity (Lamoth et al., 2020). Indeed, several studies have demonstrated that potential therapeutic compounds were ineffective under *in vitro* conditions that mimic physiological salt and serum concentrations, but were active in animal models (Dorschner et al., 2006; Myhrman et al., 2013; Maiti et al., 2014).

We could show that the activity of AFPs is influenced by the composition of the test medium (Huber et al., 2018). In high ionic strength medium, the AFPs binding to the negatively charged fungal cell membrane may be disturbed, and the susceptibility of fungi to AFPs may be missed under these conditions (Galgóczy et al., 2019; Kovács et al., 2019; Huber et al., 2020).

Therefore, more well-designed, larger-scale studies and clinical trials are necessary to integrate AFPs into existing protocols and to meet regulatory challenges (Altarac et al., 2021). It is essential to address several key parameters in the development of AFPs as pharmaceutical agents. These include pharmacokinetics and pharmacodynamics, proteolytic stability, bioavailability, tissue permeation, efficient delivery at the infection site, and the interaction with other medications. The advancement of AFPs as antifungal drugs can be achieved through the development of novel formulation strategies and drug/compound delivery systems (DDS) that employ biocompatible and biodegradable materials (Martin et al., 2015; Cuggino et al., 2019). The loading of AFPs into DDS, for example, may facilitate their metabolic and chemical stability, enhance their tissue permeation, and support their time- and site-specific delivery. The feasibility of rational design, chemical synthesis, and novel modification strategies facilitates the development of improved AFPs to meet all the requirements necessary to reach the market (Vlieghe et al., 2010).

## 6 Conclusion

The use of AFPs derived from Eurotiales as antifungal drugs demonstrates considerable promise, offering innovative solutions to address the mounting issue of fungal infections and drug resistance. Nevertheless, the clinical implementation of AFPs has not yet gained significant traction, largely due to the presence of formidable challenges, including regulatory obstacles and the necessity for integration into existing treatment protocols. To overcome these hurdles and fully exploit the potential of these molecules in clinical and agricultural contexts, it is imperative to enhance funding options to stimulate research in this scientific field and bridge the gap between academic and industrial partners. As our comprehension of protein and peptide biology and technology deepens, Eurotiales AFPs may emerge as cornerstone of antifungal therapy, offering effective and sustainable treatment alternatives.
