# Corrigendum: Navigating the fungal battlefield: cysteine-rich antifungal proteins and peptides from Eurotiales

**DOI:** 10.3389/ffunb.2024.1511041

**Published:** 2024-11-11

**Authors:** Jeanett Holzknecht, Florentine Marx

**Affiliations:** Biocenter, Institute of Molecular Biology, Innsbruck Medical University, Innsbruck, Austria

**Keywords:** antifungal proteins and peptides, fungal pathogens, fungal infection, antifungal resistance, structure-function relation, antifungal mode of action

In the published article, there were few errors in **Table 1** as published. The heading “pI at pH 7” is wrong. The correct heading is “pI”. The heading “Lys/His/Arg” is wrong. The correct heading is “Lys/Arg/His”. *Aspergillus giganteus* was listed with the strain identification number “MDH18894”. *Aspergillus giganteus* should be listed without strain identification number. The numbers 4/1/4 for Lys/Arg/His of BP from *Penicillium brevicompactum* is wrong. The correct numbers are 4/4/1. The MW 8.1 indicated for PeAfpC from *Penicillium expansum* is wrong. The correct MW is 6.7. **Table 1** appears below.

**Table 1:** A selection of representative AFPs from Eurotiales and their physicochemical properties^&^.

clade^*^	AFP	producing organism	no aa/MW^$^	pI	Lys/Arg/His	reference
AFP	AgAFP	*Aspergillus giganteus*	51/5.8	9.3	12/1/0	Wnendt et al. (1994)
BP BP BP	BP PAFC PeAfpC	*Penicillium brevicompactum* *Penicillium chrysogenum* *Penicillium expansum*	64/6.6 64/6.6 64/6.7	7.7 7.7 6.9	4/4/1 2/6/1 3/5/1	Seibold et al. (2011) Holzknecht et al. (2020) Garrigues et al. (2018)
NFAP2	NFAP2	*Neosartorya fischeri*	52/5.6	9.0	7/0/2	Tóth et al. (2016)
PAF PAF PAF PAF PAF PAF PAF	AnAfp NFAP PAF PAFB PeAfpA PeAfpA PeAfpA	*Aspergillus niger* *Neosartorya fischeri* *Penicillium chrysogenum* *Penicillium chrysogenum* *Penicillium expansum* *Penicillium expansum* *Penicillium digitatum*	58/6.5 57/6.6 55/6.3 58/6.5 57/6.6 58/6.6 58/6.6	6.6 8.9 8.9 8.8 9.5 8.3 9.1	5/2/6 11/2/1 13/0/0 8/2/6 12/3/0 9/2/6 5/5/5	Lee et al. (1999) Kovács et al. (2011) Marx et al. (1995) Huber et al. (2018) Garrigues et al. (2018) Garrigues et al. (2018) Garrigues et al. (2017a, 2017b)

^&^Physicochemical properties were calculated by protparam (www.protparam.org). ^*^AFPs are listed according to their classification by clades which are defined based on the phylogenetic tree described in Sonderegger et al. (2018). **^$^** Molecular weight of the mature protein is given in kDa.

The authors apologize for this errors and state that this does not change the scientific conclusions of the article in any way. The original article has been updated.

